# Glycodeoxycholic and deoxycholic bile acids impair recognition and spatial memory in adult mice, and reduce central CREB-BDNF signaling and cytokine expression with neuroanatomical specificity

**DOI:** 10.1080/19490976.2026.2701471

**Published:** 2026-07-11

**Authors:** Ana Garcia, Dakshat Trivedi, Daniel C. Anthony, Jonathan R. Swann, Philip W.J. Burnet

**Affiliations:** a Department of Psychiatry, University of Oxford, Oxford, UK; b School of Human Development and Health, University of Southampton, Southampton, UK; c Department of Pharmacology, University of Oxford, Oxford, UK

**Keywords:** Cognitive impairment, glutamate, inflammation, gut–brain axis

## Abstract

Emerging evidence suggests that bile acids, traditionally recognized for their role in digestion, also influence brain function and memory. This study examined the effects of two microbiota-derived secondary bile acids, deoxycholic acid (DCA) and glycodeoxycholic acid (GDCA), on memory in mice and the associated molecular mechanisms. Male and female mice received daily oral administration of DCA, GDCA, or vehicle, and spatial working and reference memory (Y-maze) and recognition memory (novel object recognition task) were assessed. After testing, gene expression and signaling activity were measured in the frontal cortex and hippocampus. Administration of GDCA after 10 d disrupted recognition memory, whereas DCA intake for 12 d impaired spatial reference memory. Neither bile acid administered for 5 d affected spatial working memory. GDCA reduced NMDA receptor subunit (GluN1, GluN2A) mRNAs and encoded protein and brain-derived neurotrophic factor (BDNF) mRNA expression and attenuated CREB signaling in the frontal cortex, which is consistent with the observed recognition memory deficit. GDCA did not alter the abundance of transcripts encoding bile acid receptors (FXR or TGR5) or their corresponding protein levels. In contrast, DCA modified the FXR and TGR5 mRNAs and proteins in a region-specific manner and decreased CREB signaling in the hippocampus, likely contributing to spatial memory deficits. In the frontal cortex, DCA increased GluA1 phosphorylation and reduced IL-1β and IL-6 expression, which may have helped preserve recognition memory. Exploratory metagenomic analysis of fecal samples showed no significant microbial differences, though subtle, non-significant functional gene changes suggested early adaptations. These findings reveal that DCA and GDCA exert distinct, receptor- and region-specific effects on cognition, identifying bile acids as modulators of microbiome–gut–brain communication.

## Introduction

Secondary bile acids are molecules produced by gut microbes through the chemical modification of primary bile acids, which are originally synthesized in the liver from cholesterol. Once secreted into the intestine, primary bile acids are transformed by specific bacterial enzymes into secondary bile acids, such as deoxycholic acid (DCA) and the glycine-conjugated form, glycodeoxycholic acid (GDCA)^1^ ^,^ ^2^. These microbial metabolites not only aid in digestion and nutrient absorption but are increasingly recognized as signaling molecules that can influence host physiology. Bile acids interact with specific receptors such as the farnesoid X receptor (FXR) and Takeda G protein-coupled receptor 5 (TGR5), which are expressed in multiple organs, including the brain.^3^ Through these receptors, bile acids may directly modulate neuroinflammatory pathways, energy homeostasis, and neurotransmission, highlighting a potential mechanism by which the gut microbiome communicates with and affects brain function.^4^ ^,^ ^5^

Recent studies have linked elevated circulating concentrations of DCA and GDCA to the pathophysiology of AD and associated cognitive impairment. For example, Nabizadeh et al.^6^ found that serum GDCA levels were positively correlated with increased cerebrospinal fluid (CSF) tau concentrations and with longitudinal changes in brain amyloid-beta (Aβ) and tau deposition, as revealed by PET imaging in individuals diagnosed with AD. Furthermore, associations between GDCA or DCA levels and cognitive impairment have been observed not only in patients with AD,^7^ ^,^ ^8^ but also in healthy individuals and those with mild cognitive impairment.^9^ Although preclinical studies suggest that elevated bile acid levels can impair cognitive function,^10^ the direct mechanisms underlying the potential neurotoxic effects of DCA and GDCA remain unclear. In particular, it is not known whether elevated concentrations of these bile acids are inherently harmful to healthy neural processes by directly activating brain FXR and TGR5.

One study demonstrated that activation of the TGR5 receptor improved spatial memory in mice with streptozotocin-induced cognitive impairment,^11^ while activation of farnesoid X receptors (FXRs) produced both anti-inflammatory and antidepressant effects in mice.^12^ These findings suggest that stimulating bile acid receptors can exert beneficial effects on brain function. However, prolonged stimulation by raised levels of bile acids or even altered expression/sensitivity of their receptors during aging may impart detrimental effects, as suggested by the aforementioned associations between biofluid bile acid levels and cognitive function. Their influence on other physiological systems may also complicate these outcomes. For example, FXR activation has been linked to the function of glutamate GluA1-containing AMPA receptors in the cortex via the inflammasome pathway,^12^ and another bile acid, chenodeoxycholic acid, has been reported to directly bind as an antagonist to glutamate NMDA receptors (NMDAR).^13^

It is therefore possible that DCA and GDCA may influence glutamate receptor signaling either directly or indirectly and thereby alter cognitive function. Given that NMDAR and GluA1-AMPA receptors activation leads to downstream activation of cAMP response element-binding protein (CREB), which can bind to BDNF promoters and regulate its expression,^14^ ^,^ ^15^ and that both CREB and BDNF can also be modulated by TGR5,^11^ it is important to investigate the potential convergence of TGR5, FXR, GluA1-AMPAR, and NMDAR in regulating CREB signaling, BDNF levels, and, therefore, cognitive function.

Despite these emerging links, the direct neurobiological actions of DCA and GDCA remain unclear. The aim of the current investigation was to explore how elevating concentrations of circulating DCA or GDCA influenced cognitive function, bile acid, and GluA1 and NMDAR subunit expression and their downstream effects. This was achieved by: (1) administering either DCA or GDCA to male and female mice and measuring their effect on recognition memory using the Novel Object Recognition (NOR) test and spatial memory (working and reference) in the Y-maze. Cognitive tests in rodents that rely on natural exploratory behavior are preferable to reward-based tasks for assessing gut–brain axis function, as the dietary restriction and food incentives required in such tasks can influence this pathway and confound the true effects of a dietary intervention^16^ and confound the true effects of a dietary intervention. Indeed, calorific restriction itself can disrupt cognitive function.^17^ (2) Evaluating the expression of NMDAR subunits, FXR, TGR5, and inflammatory cytokine (IL-1β, IL-6, and TNFα) mRNAs in the cortex and hippocampus of mice administered with DCA or GDCA. (3) Determine the levels of activated (phosphorylated) CREB and GluA1 proteins in the hippocampus and cortex of mice. An exploratory metagenomic analysis was also performed to assess whether DCA and GDCA influence gut microbial composition or functional potential. Given that bile acids can act as antimicrobial and signaling molecules shaping microbial diversity and metabolism,^1^ ^,^ ^18^ this analysis aimed to provide preliminary insight into whether exogenous bile acid administration produces detectable microbial changes. This study was not designed to test whether the cognitive effects of DCA or GDCA are mediated by the microbiome.

## Materials and methods

### Animals

All experimental procedures conformed to the requirements of the UK Animals (Scientific Procedures) Act 1986 and received approval from the University of Oxford’s Animal Welfare and Ethical Review Body. Young adult (approximately 7-week-old) male and female C57Bl/6 mice were obtained from Charles River (USA). The animals were maintained under standard laboratory conditions, including a 12-hour light/12-hour dark cycle (lights on at 07:00), an ambient temperature of 21 ± 1 °C, and a relative humidity of 50 ± 5%. Food and water were available at all times. These housing and husbandry conditions represent standard practice for animal research at the University of Oxford and have been described previously.^19^

### Experimental design

#### Bile acid administration

Mice were gavaged daily with either alkaline water (100–120 µl, pH = 8.5–9.0, *n* = 18), DCA (30 mg/kg in 100–120 µl alkaline water, *n* = 18), or GDCA (30 mg/kg in 100–120 µl alkaline water, *n* = 18) for 5 d, prior to the start of behavioral tests. The dose was chosen based on a previous study of bile acids in C57Bl/6 mice.^20^ All animals were monitored for adverse effects of bile acids, and weighed regularly to ensure that the dose of bile acids remained consistent. Bile acids were administered daily throughout the experimental period to maintain elevated circulating levels.

#### Behavioral tests

**Figure 1.**
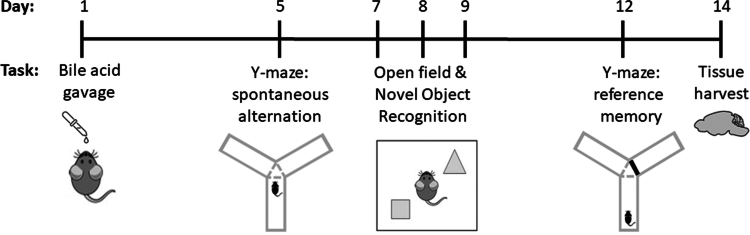
Experimental timeline for daily bile acid administration and behavioral testing in male and female mice over 14 d.

After 5 d of bile acid or vehicle administration, the mice were subjected to open field, novel object recognition and spatial memory tests (see Figure 1), which are described in detail below. This behavioral test battery has recently been described.^21^ All the mice were humanely culled 24 h after the completion of the last test.

#### Y-maze

Short-term memory tasks were run as previously described.^22^ Briefly, all the mice were handled and acclimatized to the testing room containing the Y-maze apparatus over 7 d prior to testing. To measure spontaneous alternation (spatial working memory), individual mice were placed in one arm of the Y-maze, and left to explore for 8 mins. They were then returned to their home cage, and the next mouse tested after the apparatus was cleaned with a 70% ethanol solution. An overhead video camera recorded all exploratory behavior, and videos were analyzed using the ANY-maze software (Stoeling Co., USA). The percentage of arm alternation was calculated using the formula: %Alternation = (number of alternations/[total number of arm entries-2]) x 100.

The mice were not tested again for another 7 d so that they became ‘defamiliarized’ with the Y-maze apparatus and thereby have a greater motivation to explore when testing spatial reference memory. On the test day, mice were placed in one arm (the ‘Start’ arm) of the Y-maze, which had one arm blocked. They were left to explore the two available arms for 15 min under the video camera, and then returned to their home cage. After 1 h, mice were placed in the original start arm of the Y-maze, but the previously blocked arm (‘novel’ arm) was now freely accessible. Mice were left to explore the three arms of the maze for 5  mins. Videos were analyzed to determine the number of entries into, and the time spent within, the start, novel, and third arms. The percentages of the number of novel arm visits and duration are presented and were calculated as: (Novel arm entries or duration/total number of all arm entries or duration) × 100.

### Open field and novel object recognition (NOR) tests

#### Apparatus

The testing arena for the open field was a black acrylic box measuring 30  cm high, 50  cm deep, and 50  cm wide. Exploration of the open field also served as the habituation step for the NOR task. For the latter, the objects used were Falcon tissue culture flasks filled with sand (9.5  cm high, 2.5  cm deep, and 5.5  cm wide, made of transparent plastic with a red bottle cap) and 50  ml self-standing centrifuge tubes filled with sand (measuring 2.91  cm × 11.49  cm, made of transparent plastic with a red bottle cap). Three copies of each object were made: two were randomly used for the familiarization session, and the third was used for the test session. The NOR test was run as previously described.^23^

#### Procedure

*Open field/NOR habituation:* Twenty-four hours before the familiarization session, mice were individually placed in the empty, open field, facing the wall closest to the experimenter, and allowed to explore for 5 min. After returning the mouse to its home cage, the open field box was cleaned with 70% (vol/vol) ethanol to minimize olfactory cues. All activity in the open field was recorded with an overhead video camera. The times spent in the outer and inner sectors of the open field and total distance traveled were assessed using the ANY-maze program.

*NOR Familiarization session:* Twenty-four hours after habituation, the two identical objects (either Falcon tissue culture flasks or centrifuge tubes) were placed in the open field, 5 cm away from the walls. The pair of objects was randomized between each mouse and each group tested. The mouse was placed in the open field facing the wall opposite the objects and allowed to explore for 10 min. After returning the mouse to its home cage, the objects and experimental apparatus were cleaned with 70% (vol/vol) ethanol and dried with paper towels.

*NOR Test session*: Twenty-four hours after the familiarization session, the two familiar objects were replaced. One object was substituted with a triplicate copy (to ensure there were no residual olfactory cues on the previously used object), and the other with a novel object. The new set of different objects was positioned in the same spot, 5 cm away from the walls. The position of the novel object (left or right) was randomized between each mouse and each group tested. The mouse was placed in the open field facing the wall opposite the objects and allowed to explore for 10 min before being returned to its home cage. The objects and experimental apparatus were then cleaned with 70% (vol/vol) ethanol and dried with paper towels before the next animal was tested. The total time each animal spent exploring the novel and familiar objects was digitally recorded and analyzed using ANY-maze.

*Calculations:* Discrimination (D) was calculated as the difference between time spent exploring novel and familiar objects, during the test phase, i.e., the exploration time devoted to the novel object (TN) minus the time dedicated to the familiar object (TF), [D = (TN – TF)]. Discrimination index (DI), the standard metric for assessing recognition memory, was calculated using the formula: DI = D/(TN + TF). The results vary between +1 and -1 (a positive score indicates more time spent with the novel object, a negative score indicates more time spent with the familiar object, and a zero score indicates a null preference).

### Transcript and protein measurements

#### Tissue collection

All animals were culled by cervical dislocation, brains were removed, and the frontal cortex and hippocampus were dissected and stored at -80 °C. These brain areas were chosen as regions involved in the modulation of cognitive function.^24^ ^,^ ^25^

#### RNA extraction and quantitative PCR (QPCR)

The following procedures were carried out as previously described.^19^ Total RNA was extracted from all tissue samples using TRIzol reagent (Thermo Fisher Scientific, UK) in accordance with the manufacturer’s protocol. The resulting RNA was resuspended in RNase-free water, and its concentration was quantified with the Qubit RNA BR assay (Thermo Fisher Scientific). For each sample, 1  μg of RNA was treated with DNase (Promega) and then reverse-transcribed into cDNA using the High-Capacity cDNA Reverse Transcription Kit (Thermo Fisher Scientific, UK). To confirm the absence of genomic DNA contamination, a negative control reaction was prepared using 1  μg of RNA in the absence of reverse transcriptase.

Quantitative PCR was performed in triplicate in a final reaction volume of 12  μl consisting of 6  μl of 2 × PowerTrack SYBR Green Master Mix (Applied Biosystems™, Thermo Fisher Scientific, UK), 1  μl of each gene-specific forward and reverse primer (10 pmol/μl), and 4  μl of the cDNA template. The primers used were as follows: FXR (forward: 5′-TGTGAGGGCTGCAAAGGTTT-3′; reverse: 5′-ACATCCCCATCTCTGCAC-3′), TGR5 (forward: 5′-CTTCTCTCTGTCCGCGTGTT-3′; reverse: 5′-GCCAGGGTTGAGGGTACATC-3′), GluN1 (forward: 5′-ATCATCCTGCTGGTCAGCGA-3′; reverse: 5′-AGCAGAGCCGTCACATTCTT-3′), GluN2A (forward: 5′-GCTTTCCTTGAACCCTTCAG-3′; reverse: 5′-AACTTAGCCAAAGGGAAAGCTCCCGA-3′), GluN2B (forward: 5′-TTGGTGAGGTGGTCATGAAG-3′; reverse: 5′-ACCTTCTGCCTTCTTAGAGCC-3′), BDNF (forward: 5′-GCGCCCATGAAAGAAGTAAA-3′; reverse: 5′-TCGTCAGACCTCTCGAACCT-3′), and GAPDH (forward: 5′-GTATTGGGCGCCTGGTCACC-3′; reverse: 5′-CGCTCCTGGAAGATGGTGATGG-3′). Gene expression was normalized to GAPDH levels, and relative fold changes were calculated using the 2^−^ΔΔCt method as described by Livak and Schmittgen.^26^

#### Western blotting

Immunoblotting was performed in accordance with previously published methods^19^ using hippocampal and frontal cortex samples from four male and four female mice per experimental group. Tissue samples were homogenized in RIPA lysis buffer (Sigma–Aldrich, UK) supplemented with 0.1% (v/v) protease inhibitor cocktail. The protein content of each homogenate was quantified using the Bradford protein assay (Bio-Rad Laboratories, CA, USA). An equal amount of protein (40  μg) from each sample was loaded onto precast AnyKD Mini-Protean TGX gels (Bio-Rad) and separated by SDS–PAGE using 1 × running buffer (25 mM Tris base, 250  mM glycine, 0.1% SDS in dH₂O). Following electrophoresis, proteins were transferred overnight onto polyvinylidene difluoride (PVDF) membranes by electroblotting in 1 ×  transfer buffer (25 mM Tris base, 192 mM glycine, 20% methanol in dH₂O). The membranes were then briefly immersed in methanol for activation and blocked for 1 hour at room temperature in blocking solution consisting of 0.1% PBS-Tween 20 (PBS-T) containing 5% skimmed milk.

Primary and secondary antibodies were diluted in 0.1% PBS-T supplemented with 2% milk and incubated with the membranes sequentially for 1 h and 40 min, respectively, with three 20-min washes in PBS-T between incubations. The primary antibodies used were: anti-FXR (1:1000, Abcam UK Ltd.), anti-TGR5 (1:1500, Abcam UK Ltd.), anti-GluN1 (1:1000, Thermo Fisher Scientific Inc, USA), anti-GluN2A (1:1000, Thermo Fisher Scientific Inc, USA), anti-GluN2B (1:1000, Thermo Fisher Scientific Inc, USA), anti-phospho-CREB (Ser133; 1:2000, Abcam UK Ltd), anti-CREB (1:2000, Merck Life Sciences, UK), anti-phospho-GluA1 (Ser845; 1:1000, Abcam Ltd, UK), anti-GluA1 (1:1000, Abcam Ltd, UK), and anti-*β*-actin (1:5000, Merck Life Science UK Ltd). Horseradish peroxidase–conjugated goat anti-rabbit (1:10,000, Thermo Fisher Scientific, UK) and goat anti-mouse (1:5,000, Thermo Fisher Scientific, UK) antibodies were used as secondary antibodies. Immunoreactive bands were detected using enhanced chemiluminescence (ECL™ Prime Western Blotting System, Merck Life Science UK Ltd) and visualized by exposure to Hyperfilm™ ECL™ (Merck Life Science UK Ltd) for varying durations. Band intensity was quantified with an AlphaImager 3400, ensuring that film exposures were within the linear (non-saturated) range. Relative immunoreactivity was normalized to *β*-actin, which served as the internal loading control. All data were then expressed as a fold-change relative to the respective controls.

### Targeted quantification of bile acids

A targeted LC‒MS/MS technique using multiple reaction monitoring (MRM) transitions tailored for specific bile acids was employed for measuring bile acid concentrations in mice. The approach utilized bile acid reference standards and deuterated bile acid forms as internal standards (IS). Calibration curves were constructed over a concentration range of 5 nM to 8000 nM. Samples were deproteinated, and bile acids were extracted using a mix of aqueous and organic solvents, as detailed in the Supplementary information. An Acquity UPLC coupled to a Waters Xevo TQ-XS triple quadrupole mass spectrometer (MS) was used for bile acid quantification. Details of mobile phases, elution gradient, and flow rates used for bile acid separation are provided in the supplementary information. Briefly, the mass spectrometer was operated in negative electrospray ionization (-ESI) mode using nitrogen as the desolvation gas and argon as the collision gas. Detailed MS instrument settings, along with the calibration and quantification procedures, are also detailed in the Supplementary material.

### Metagenomic analysis of mouse fecal samples

Fecal pellets were collected from the mice during brain tissue harvest and stored at −80 °C. Two fecal pellets from six randomly selected mice per group were sent to Transnetyx, Inc. (Cordova, TN, USA) for sequencing. Sample processing and analysis are described in the Supplementary material.

### Data analysis

Statistical analysis of all behavioral and statistical data was performed using SPSS (version 28). The skewness of data was explored with the Shapiro‒Wilk test, and identified outliers (≥3SD) were removed prior to analysis. All results are presented as medians and interquartile ranges with minimum and maximum data points. Behavioral and gene expression data were subjected to multivariate analyzes to test for treatment (water, bile acid) x gender (male, female) interactions, and the effects of treatment or gender alone. For the open field, a treatment x gender x zone (inner, outer) interaction was explored. Where significant interactions/effects were detected (*p* < 0.05), post hoc pairwise comparisons (with Bonferroni correction) were performed to reveal group differences. All western blot data were analyzed with One-way ANOVA with post hoc Tukey tests when group differences (*p* < 0.05) were detected.

Metagenomic data were analyzed in R (version 4.5.1) to evaluate observational richness, alpha and beta diversity, and to identify changes in taxonomic and functional gene composition between groups (see Supplementary material).

## Results

### GDCA impairs recognition memory without altering locomotor activity

**Figure 2.**
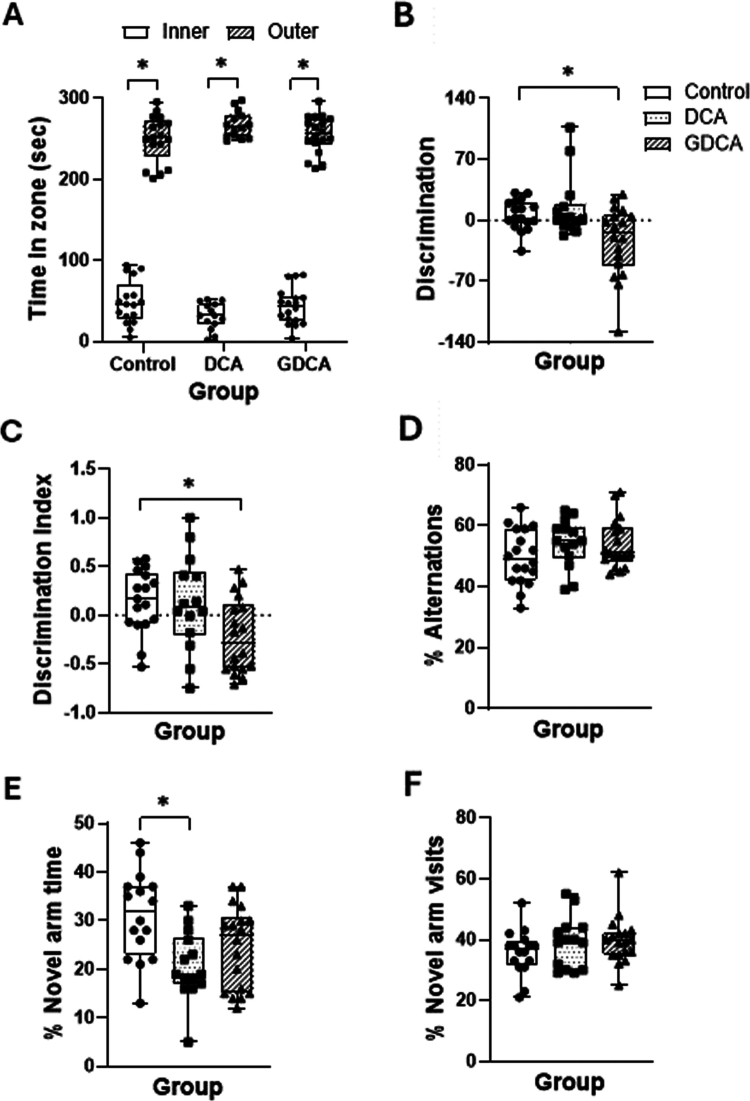
The effects of DCA and GDCA on performance in the open field and NOR task (A, B, C) and spatial memory in the Y-maze (D, E, F). (A) Oral administration of DCA or GDCA to mice did not influence behavior in the open field compared to controls. Mice in each group spent significantly more time in the outer zone than in the inner zone (*p* < 0.05). (B) Intake of GDCA, but not DCA, significantly reduced novel and familiar object discrimination compared to controls in the NOR task. (C) GDCA administration reduced the Discrimination Index (recognition memory) compared to the control group. Recognition memory was not affected by DCA intake. (D) Bile acid administration to mice did not affect spontaneous alternations (spatial working memory) in the Y-maze. (E) Administration of DCA, but not GDCA, reduced the percentage of time spent in the novel arm, indicating an impairment of spatial reference memory. (F) Intake of bile acids did not alter the percentage of visits to the novel arm (*p* > 0.05). There were no treatment-by-gender interactions observed in any of the behavioral parameters. Data are presented as median and interquartile range with min/max data points, and examined using multivariate analysis (NOR or Y-maze parameters analyzed separately), with post hoc pairwise comparisons when there was a significant interaction/effect. *p < 0.05; *n* = 7–9 mice/gender/group; *n* = 15–18 mice/group.

There were no treatment x gender interactions (*F*_1,43_ < 2.5, *p* > 0.05) or effects of treatment (*p* > 0.05) on the number of times mice spent in the outer zone or inner zone of the open field (Figure 2A). In all groups, mice spent more time in the outer zone than in the inner zone of the open field (*p* < 0.05). In the NOR task, multivariate analysis revealed that in the absence of a treatment x gender interaction (*F*_1,43_ < 2.5, *p* > 0.05), there was a significant effect of treatment on Discrimination (*F*_1,43_ = 7.02, *p* = 0.002) and Discrimination Index (*F*_1,43_ = 4.67, *p* = 0.015). Pairwise comparisons with controls showed that the GDCA group had lower Discrimination (Figure 2B, *p* = 0.043) and Discrimination Index (Figure 2C, *p* = 0.023) values.

### DCA selectively disrupts spatial reference memory in the Y-maze

The intake of bile acids did not affect spontaneous alternation in the Y-maze (Figure 2D). However, in the assessment of reference memory, there was a significant effect of treatment on the time spent in the Novel arm (*F*_1,42_ = 6.08, *p* = 0.005), with pairwise comparisons revealing that the duration of visits to this arm was lower in the DCA group than in controls (Figure 2E, *p* = 0.004). There were no effects of bile acids on the number of visits to the Novel arm (*F*_1,42_ < 2.5, *p* > 0.05, Figure 2F). A treatment x gender interaction (*F*_1,43_ < 2.5, *p* > 0.05) was not detected for spatial working memory or parameters of spatial reference memory (*p* > 0.05).

### DCA and GDCA reduce FXR, TGR5, NMDA receptor mRNAs and protein, and BDNF mRNA expression with regional specificity

**Figure 3.**
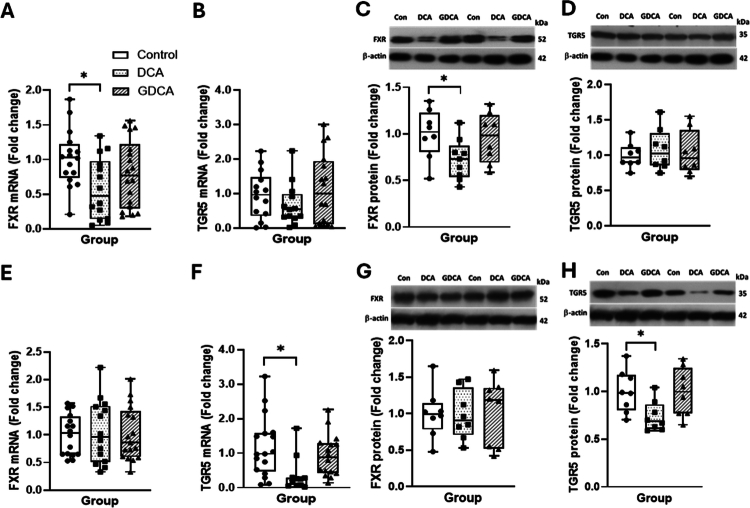
Bile acid receptor (FXR, TGR5) mRNA expression (A, B, E, F) and protein (immunoreactivity in western blots, C, D, G, H) levels in the pre-frontal cortex (A–D) and hippocampus (E-H) of male and female mice administered with DCA or GDCA. (A) Intake of DCA, but not GDCA, reduced FXR mRNA abundance in the cortex compared to controls. (B) Neither DCA nor GDCA intake by mice affected the expression of TGR5 mRNA. (C) *Top panel:* Representative immunoblots of FXR in the frontal cortex compared to *β*-actin. *Bottom panel:* FXR:β-actin gray density ratios expressed as a fold change relative to control values. DCA administration reduced cortical FXR protein compared to controls, whereas GDCA was without effect. (D) *Top panel:* Representative immunoblots of TGR5 in the frontal cortex compared to *β*-actin. *Bottom panel:* TGR5 protein levels expressed as the fold change relative to controls in the cortex were not influenced by the administration of either DCA or GDCA. (E) Administration of bile acids did not affect the abundance of FXR mRNA in the hippocampus. (F) Ingestion of DCA significantly reduced hippocampal TGR5 mRNA expression in the hippocampus. This effect was not observed following GDCA administration. (G) *Top panel:* Representative immunoblots of FXR in the hippocampus compared to *β*-actin. *Bottom panel*: FXR:β-actin gray density ratios expressed as a fold change compared to control values. Bile acid intake did not alter FXR protein in the hippocampus. (H) *Top panel:* Representative immunoblots of TGR5 in the hippocampus compared to *β*-actin. *Bottom panel*: TGR5:β-actin gray density ratios expressed as a fold change compared to control values. A significant reduction of TGR5 protein levels was observed in the hippocampus of mice administered with DCA, but not with GDCA. No treatment-by-gender interaction was observed for any of the expressed genes and proteins analyzed. Data are presented as the median and interquartile range with min/max data points of fold changes compared to control mice. All mRNA expression data were examined using multivariate analysis (frontal cortex and hippocampus separately) with post hoc pairwise comparisons when there was a significant interaction/effect. *p < 0.05, *n* = 7–9 mice/gender/group; *n* = 15–18 mice/group. The Western blot data were analyzed with one-way ANOVA with post hoc Tukey tests when group differences (*p* < 0.05) were detected. *p < 0.05, compared to controls; *n* = 4 mice/gender/group; *n* = 8 mice/group. Con = controls, DCA = deoxycholic acid, GDCA = glycodeoxycholic acid, kDa = relative molecular weight of immunoreactive bands.

Analysis of mRNA expression in both the frontal cortex and the hippocampus did not reveal a treatment x gender interaction (*p* > 0.05). In the frontal cortex, there was a significant group difference in FXR expression (*F*_1,36_ = 3.68, *p* = 0.037), with pairwise comparisons revealing that FXR mRNA abundance was lower in the DCA group compared to the control group (Figure 3A). The expression of TGR5 mRNA in the frontal cortex was similar in all three groups (Figure 3B). Analysis of cortical FXR protein levels, measured as fold change relative to controls and quantified as FXR immunoreactivity normalized to *β*-actin abundance on Western blots, revealed a significant group difference (*F*_2,21_ = 4.02, *p* = 0.033), with *posthoc* Tukey testing demonstrating a significant reduction in FXR protein in the DCA group compared with controls (Figure 3C). The levels of TGR5 protein in the frontal cortex were similar in all three groups (Figure 3D).

Bile acid administration did not affect FXR mRNA in the hippocampus (Figure 3E), but a significant effect of treatment was detected for TGR5 mRNA abundance (*F*_1,36_ = 3.51, *p* = 0.041). Post hoc analysis showed lower TGR5 mRNA expression in the DCA group compared to the control group (Figure 3F). Consistent with the encoding transcript expression, FXR protein in the hippocampus was not affected by the administration of DCA or GDCA (Figure 3G). However, a main effect of group was detected (*F*_2,21_ = 4.22, *p* = 0.029), with *post hoc* analysis revealing that TGR5 protein was significantly lower than controls (Figure 3H).

**Figure 4.**
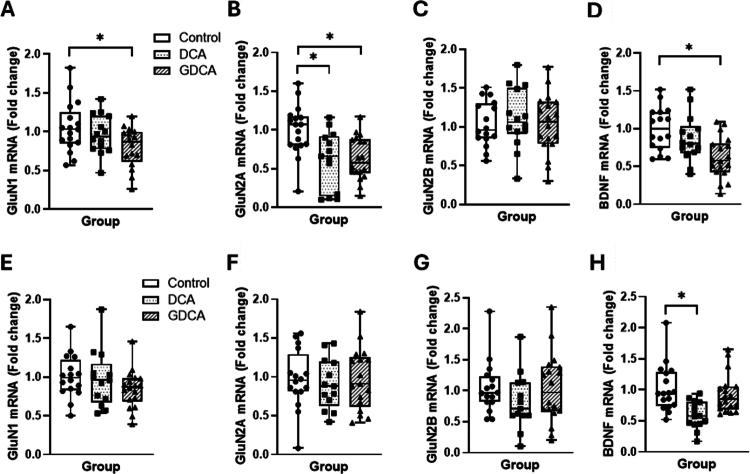
The effect of bile acid administration on the expression of mRNAs encoding NMDA receptor subunits (GluN1, GluN2A, and GluN2B) and BDNF in the frontal cortex (A, B, C, D) and hippocampus (E, F, G, H) of male and female mice. (A) Ingestion of GDCA, but not DCA, reduced GluN1 mRNA abundance compared to controls. (B) Administration of GDCA, but not DCA, reduced GluN2A mRNA abundance. (C) There were no significant effects of bile acid administration on GluN2B gene expression. (D) Ingestion of GDCA significantly reduced BDNF mRNA expression, which remained unaltered following the administration of DCA (*p* > 0.05). (E, F, G) Bile acid intake did not affect the expression of transcripts encoding NMDA receptor subunits in the mouse hippocampus. (H) Intake of DCA, but not GDCA, reduced the expression of BDNF mRNA. No treatment-by-gender interactions were observed among the expressed genes analyzed. Data are presented as median and interquartile range with min/max data points of fold changes compared to control mice. All data were examined with multivariate analysis and post hoc pairwise comparisons when there was a significant interaction/effect. *p < 0.05; *n* = 7–9 mice/gender/group; *n* = 15–18 mice/group.

In the frontal cortex, significant group differences were also detected for GluN1 (*F*_1,36_ = 3.89, *p* = 0.031), GluN2A (*F*_1,36_ = 4.08, *p* = 0.027), and BDNF (*F*_1,36_ = 5.51, *p* = 0.009) transcripts. Pairwise analysis revealed that GluN1 mRNA abundance in the GDCA group was significantly lower compared to controls (Figure 4A), and GluN2A mRNA abundance in both DCA and GDCA groups was significantly lower than in controls (Figure 4B). The expression of Gun2B mRNA was not altered by the administration of bile acids (Figure 4C). The abundance of BDNF mRNA was significantly lower in the GDCA group compared to controls (Figure 4D).

In the hippocampus, the administration of bile acids did not alter GluN1, GluN2A, or GluN2B gene expression (Figure 4E, F, G). However, there were significant group differences in BDNF expression (*F*_1,44_ = 3.89, *p* = 0.030), with post hoc analysis revealing that BDNF mRNA abundance was lower in the DCA group compared to controls (Figure 4H).

**Figure 5.**
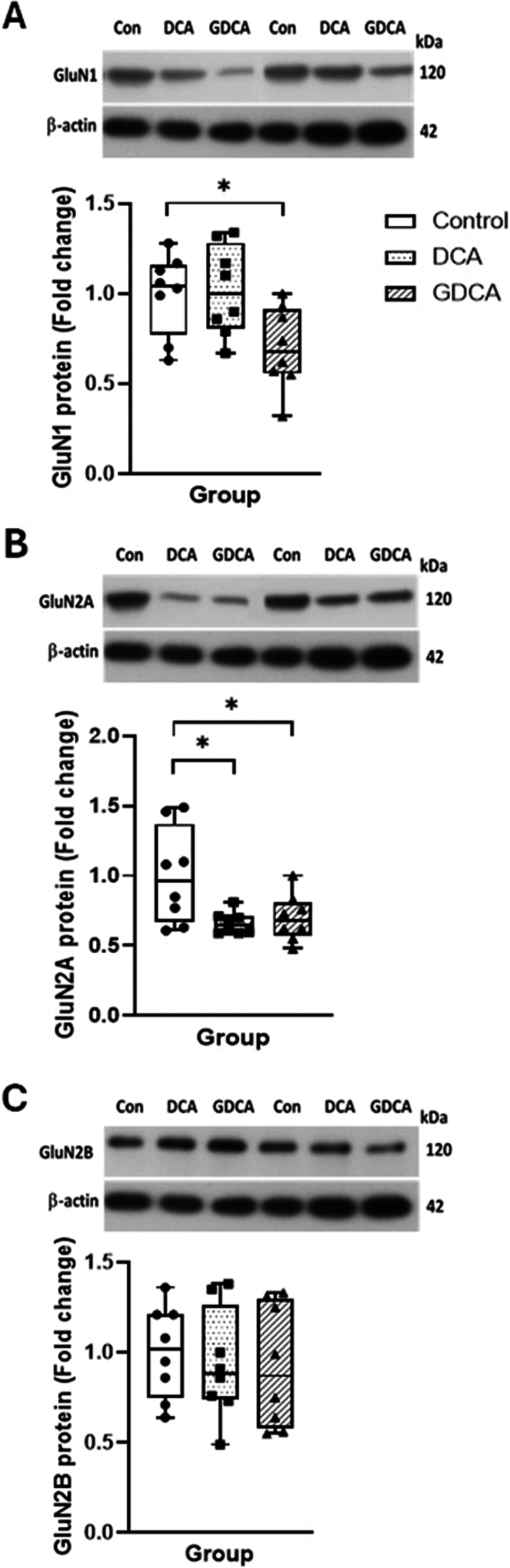
Western blot analysis of NMDA receptor subunits in the frontal cortex of bile acid administered male and female mice. (A) *Top panel*: Representative immunoblots of GluN1 and *β*-actin. *Bottom panel*: GluN1:β-actin gray density ratios expressed as a fold change compared to control values. GluN1 protein was significantly reduced compared to controls after the intake of GDCA but not DCA. (B) *Top panel*: Representative immunoblots of GluN2A and *β*-actin. *Bottom panel*: GluN2A:β-actin gray density ratios expressed as a fold change compared to control values. Administration of DCA or GDCA reduced GluN2A protein relative to controls. (C) *Top panel*: Representative immunoblots of GluN2B and *β*-actin. *Bottom panel*: GluN2B:β-actin gray density ratios expressed as a fold change compared to control values. Compared to controls, ingestion of either DCA or GDCA did not alter GluN2B protein. All data are presented as median and interquartile range with min/max data points, and analyzed with One-way ANOVA with post hoc Turkey tests when group differences (*p* < 0.05) were detected. *p < 0.05, compared to controls; *n* = 4 mice/gender/group; *n* = 8 mice/group. Con = controls, DCA = Deoxycholic acid, GDCA = glycodeoxycholic acid, kDa = relative molecular weight of immunoreactive bands.

Western blot analysis of NMDA receptor subunit proteins in the frontal cortex, where changes in encoding transcripts were detected, revealed group differences in GluN1 (*F*_2,21_ = 4.68, *p* = 0.021) and GluN2A (*F*_2,21_ = 5.38, *p* = 0.013), but not GluN2B (*p* > 0.05). Subsequent *posthoc* Tukey tests showed that relative to controls, GluN1 protein levels were significantly reduced in the GDCA group (Figure 5A), whereas GluN2A protein was reduced in both DCA and GDCA groups (Figure 5B). Similar levels of GluN2B protein were observed in all groups (Figure 5C).

The effects of bile acids on inflammatory cytokine expression in the frontal cortex and hippocampus of mice are summarized in Table 1. In the frontal cortex, significant group differences were detected for IL-1β (*F*_1,36_ = 3.73, *p* = 0.034) and IL-6 (*F*_1,36_ = 4.82, *p* = 0.014) transcripts. Pairwise analysis revealed that the abundance of mRNAs encoding these cytokines was significantly (*p* < 0.05) lower in the DCA group compared to controls. The expression of TNFα mRNA in the frontal cortex was not significantly altered by the administration of bile acids. Group differences in the expression of IL-1β, IL-6, and TNFα, were not detected in the hippocampus (*F*_1,36_ < 2.5, *p* > 0.05).

**Table 1.** The effect of bile acid administration on cytokine mRNA expression measured by QPCR, in the prefrontal cortex and hippocampus of male and female mice. All data from each area were analyzed in a multivariate analysis. There were no Treatment x Sex interactions, only an effect of treatment. *p < 0.05 compared to the control group in a pairwise comparison; *n* = 16–18 mice/group, 8–9 mice/gender.

	GROUP		
*Brain Region*	Control	DCA	GDCA
Cytokine mRNA	Fold Change (Mean + SEM)	Fold Change (Mean + SEM)	Fold Change (Mean + SEM)
*Prefrontal cortex*			
IL1β	1.00 ± 0.08	0.51 ± 0.08*	0.84 ± 0.12
IL6	1.00 ± 0.09	0.59 ± 0.09*	0.76 ± 0.11
TNFα	1.00 ± 0.14	0.77 ± 0.07	0.95 ± 0.15
IL10	1.00 ± 0.11	1.11 ± 0.17	1.14 ± 0.13
*Hippocampus*			
IL1β	1.00 ± 0.07	0.86 ± 0.14	0.87 ± 0.05
IL6	1.00 ± 0.09	0.99 ± 0.13	1.05 ± 0.11
TNFα	1.00 ± 0.09	1.13 ± 0.21	1.40 ± 0.11
IL10	1.00 ± 0.12	1.02 ± 0.12	1.01 ± 0.12

### DCA and GDCA differentially affect cortical CREB and GluA1 phosphorylation

**Figure 6.**
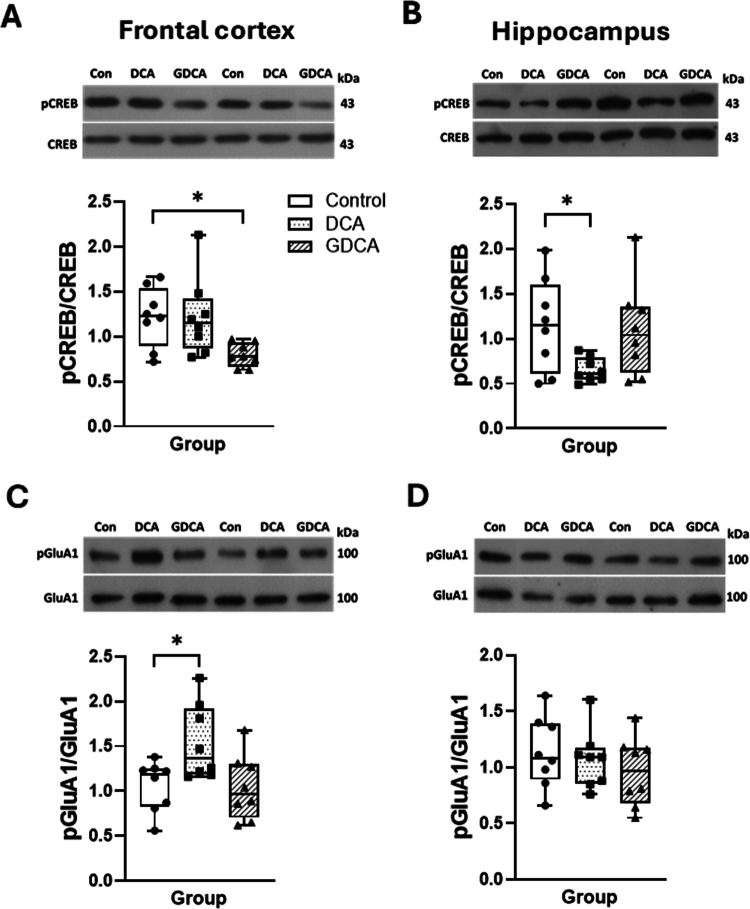
Western blot analysis of ERK1/2 signaling and AMPA receptor subunit GluA1 activation in the frontal cortex (A, C) and hippocampus (B, D) of male and female mice following bile acid administration. (A) *Top panel*: Representative immunoblots showing phosphorylated-Ser133-CREB (pCREB) and total CREB in the frontal cortex. The size of immunoreactive bands is indicated as kDa on the right of the blots. *Bottom panel*: the ratios of pCREB to total CREB immunoreactivity were significantly reduced compared to controls after the intake of GDCA but not DCA. (B) *Top panel*: Representative immunoblots showing pCREB and total CREB in the hippocampus. *Bottom panel*: Administration of DCA reduced pCREB/CREB ratios compared to controls. GDCA intake was without effect. (C) *Top panel*: Representative immunoblots of phosphorylated-Ser845-GluA1 (pGluA1) and total GluA1 in the frontal cortex. *Bottom panel*: Compared to controls, ingestion of DCA, but not GDCA, significantly elevated pGluA1/GluA1 ratios. (D) *Top panel*: Representative immunoblots showing pGluA1 and GluA1 in the hippocampus. *Bottom panel*: the administration of bile acids did not alter pGluA1/GluA1 ratios. All data are presented as median and interquartile range with min/max data points, and analyzed with One-way ANOVA with post hoc Turkey tests when group differences (*p* < 0.05) were detected. *p < 0.05, compared to controls; *n* = 4 mice/gender/group; *n* = 8 mice/group. Con = controls, DCA = Deoxycholic acid, GDCA = glycodeoxycholic acid.

One-way ANOVA demonstrated significant group differences in the phosphorylated/total ratios of CREB (*F*_2,21_ = 4.67, *p* = 0.021) and GluA1 (*F*_2,21_ = 5.03, *p* = 0.016) in the frontal cortex. Post hoc analysis revealed that CREB phosphorylation was lower in the GDCA group than in controls (Figure 6A), whereas GluA1 phosphorylation was greater in the DCA group than in controls (Figure 6C). In the hippocampus, a significant group difference was detected in the CREB phosphorylated/total ratio (*F*_2,21_ = 3.99, *p* = 0.034), with post hoc tests revealing lower levels of CREB phosphorylation in the DCA group than in controls (Figure 6B). The administration of bile acids did not affect GluA1 phosphorylation in the hippocampus (Figure 6D).

### DCA and GDCA elevate circulating bile acid levels but are undetectable in the brain

Administration of DCA and GDCA to mice resulted in elevated circulating concentrations of these bile acids 2 h after dosing (Table S1). Levels in the brain were very low, approaching the detection limit under these conditions (data not shown). Bile acid administration also increased circulating cholic acid levels, and GDCA further led to an elevation in DCA concentrations (Table S1).

### Fecal microbiota composition remains stable after DCA and GDCA exposure

**Figure 7.**
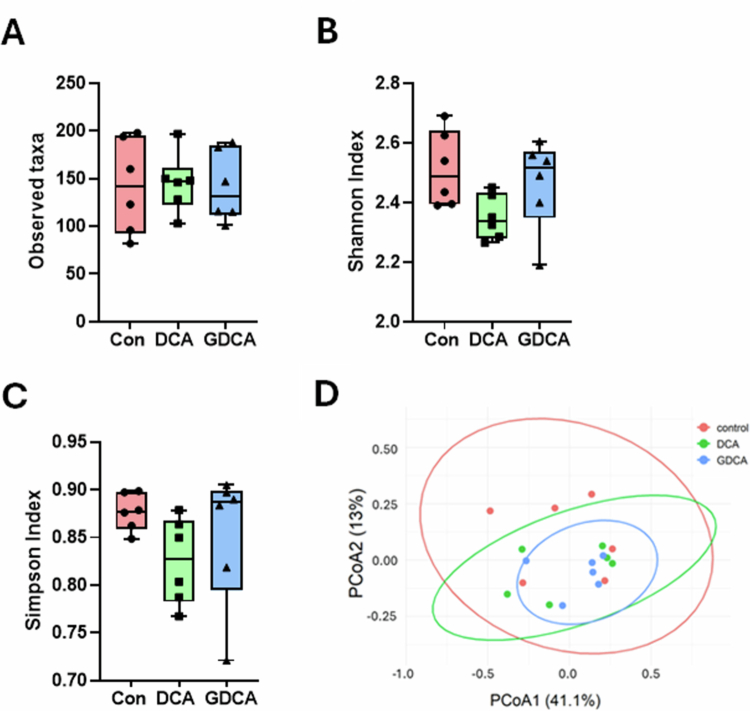
Effects of DCA and GDCA on gut microbial diversity and composition. Alpha diversity indices, including (A) Observed richness, (B) Shannon Index, and (C) Simpson Index of microbial communities in control (Con), DCA-treated, and GDCA-treated groups. All data are presented as median and interquartile range with min/max data points and analyzed with the Kruskal–Wallis test. (D) Principal Coordinates Analysis (PCoA) plot based on Bray–Curtis dissimilarity showing beta diversity of microbial communities among the three groups. Each point represents an individual sample, and ellipses indicate 95% confidence intervals for each group. Axes represent the percentage of variation explained by the first two principal coordinates.

Alpha-diversity analyzes of fecal 16S sequences in all three groups (Figure 7A, B, C) did not show significant differences in observed richness, Shannon, or Simpson indices among groups (Kruskal–Wallis H < 1.0, *df* = 2, *p* > 0.05 for all comparisons; *n* = 6 per group). Principal coordinates analysis (PCoA) based on Bray–Curtis dissimilarity showed partial overlap among groups, indicating broadly similar microbial community compositions (Figure 7D). PERMANOVA confirmed no significant group effect (*F*_2,15_ = 1.07, *R* ^2^ = 0.125, *p* = 0.35).

**Figure 8.**
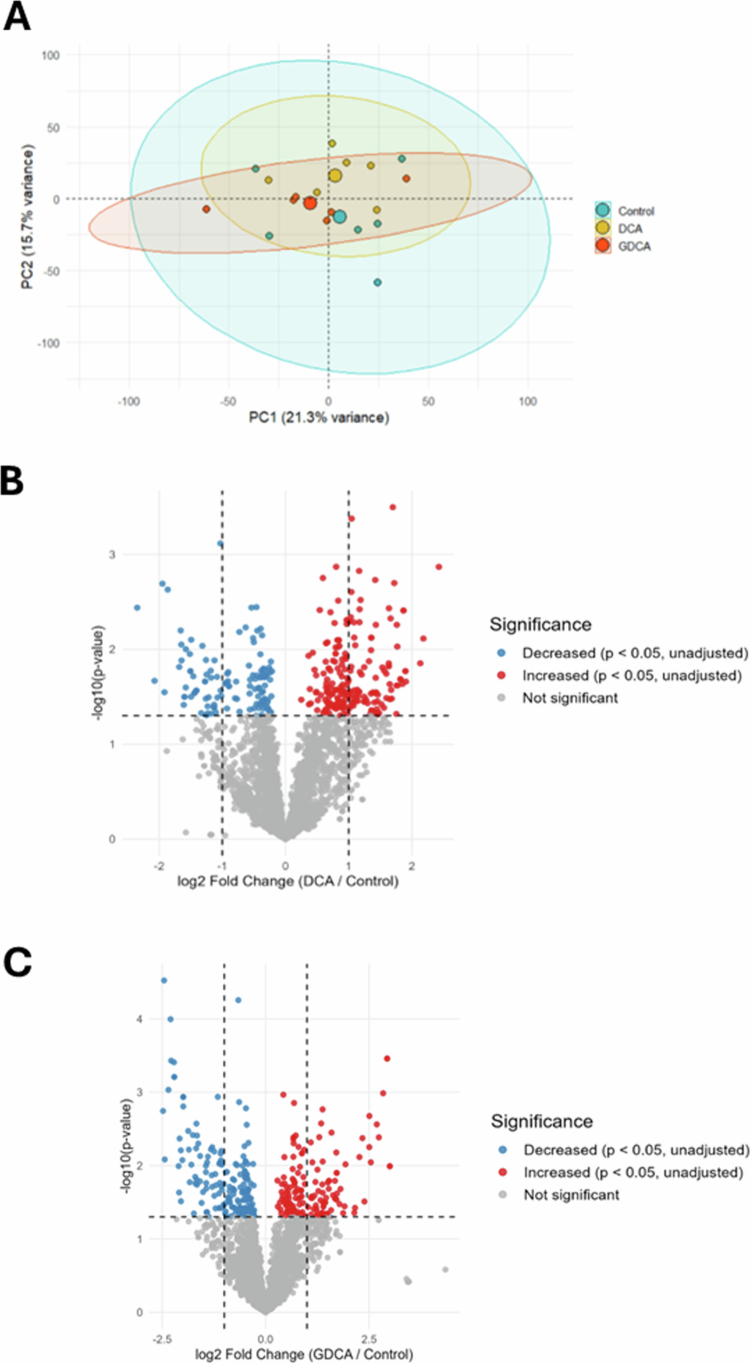
Impact of DCA and GDCA on gut microbial community structure and differential abundance of taxa. (A) Principal Component Analysis (PCA) plot based on genus-level microbial composition showing separation among Control (blue), DCA-treated (orange), and GDCA-treated (yellow) groups. Each point represents an individual sample, and shaded ellipses indicate 95% confidence intervals for each group. PC1 and PC2 represent the first and second principal components, respectively, explaining 21.3% and 17.5% of the variance. (B) Volcano plot displaying differential abundance of microbial genera between DCA and Control groups. Each dot represents a genus; red indicates significantly increased abundance (*p* < 0.05, unadjusted), blue indicates significantly decreased abundance, and gray indicates no significant change. (C) Volcano plot showing differential abundance of microbial genera between GDCA and Control groups. None of the observed significant changes survived FDR correction.

Exploratory analysis of microbial functional gene profiles (expressed as copies per million, CPM) showed partial separation between groups in the PCA ordination, suggesting subtle differences in microbial functional potential (Figure 8A). PERMANOVA indicated a modest group effect that did not reach statistical significance (*F*_2,15_ = 2.50, *p* = 0.075), with group assignment explaining approximately 25% of the total variance (*R* ^2^ = 0.25).

To further explore functional changes associated with bile acid exposure, differential abundance analyzes were performed comparing DCA and GDCA groups to controls. Volcano plots (Figure 8B, C) were generated to visualize gene-level differences in functional profiles, highlighting both the magnitude (log₂ fold change) and significance (*p*-value) of changes in functional gene abundance. Statistical comparisons were conducted using pairwise non-parametric tests (Wilcoxon rank-sum test) on CPM values. Several functional genes appeared with significant increases or decreases at an unadjusted *p* < 0.05 in both the DCA and GDCA groups relative to controls. However, none of these differences remained significant after false discovery rate (FDR) correction. Given that GDCA administration increased circulating DCA concentrations, we examined microbial bile salt hydrolase (BSH) gene abundance, the primary enzymes responsible for bile acid deconjugation. Total BSH gene abundance showed a trend toward differences between groups (Kruskal–Wallis H = 5.099, df = 2, *p* = 0.078; Figure S1A). Taxonomic analysis revealed that BSH activity was predominantly contributed by *Bacteroides spp*. (Figure S1B), highlighting the potential role of these microbes in modulating circulating bile acid composition.

To complement this targeted analysis, we also performed an untargeted analysis of microbial hydrolases to assess whether other hydrolase activities, which could also potentially deconjugate bile acids, were affected by bile acid intake. This analysis identified sulfuric hydrolases as the most significant non-BSH hit, though nominal differences between groups did not survive FDR correction (see Table S2, Figure S2, Supplementary material).

## Discussion

This study investigated the effects of two microbiota-derived, secondary bile acids, DCA and GDCA, on cognitive performance in mice and on the underlying molecular signaling pathways. Building on previous findings that link DCA and GDCA to age- and dementia-related cognitive decline, we demonstrated that DCA impaired spatial reference memory, whereas GDCA disrupted recognition memory in both male and female mice. These cognitive deficits appear to arise from selective modulation or disruption of central bile acid and glutamate receptors within specific brain regions. In the frontal cortex, GDCA reduced the expression of mRNAs encoding NMDA receptor subunits (GluN1, GluN2A) and BDNF, in addition to CREB signaling activity. However, DCA decreased the expression of FXR and GluN2A mRNAs, increased GluA1 activation, but did not affect BDNF expression or CREB signaling. In the hippocampus, DCA lowered TGR5 mRNA expression and CREB signaling, while GDCA had no observable effect. These findings suggest that DCA and GDCA may contribute to cognitive decline through distinct region- and receptor-specific mechanisms.

Recognition memory is a cognitive domain commonly impaired in dementia.^27^ Our findings support the notion that elevated circulating levels of GDCA contribute to cognitive impairment,^8^ and provide the first evidence that this impairment is specific to recognition memory. In mice, optimal functioning of the frontal cortex and NMDARs therein is essential for intact recognition memory and has been demonstrated using the NOR task.^28^ ^,^ ^29^ The molecular changes we have observed in the frontal cortex are indicative of disrupted glutamatergic neurotransmission, which is consistent with the deficits in NOR performance. Importantly, GDCA did not alter FXR or TGR5 mRNAs and encoded proteins, suggesting that its effects were independent of these bile acid receptors. The present findings instead suggest that GDCA may more directly influence NMDAR-associated signaling pathways. Although unconjugated DCA is generally considered more hydrophobic and membrane-disruptive than GDCA,^30^ both bile acids reduced cortical GluN2A expression in the present study, suggesting that differences in membrane-disrupting activity alone are unlikely to explain their distinct cognitive and signaling effects. One possibility is that GDCA interacts with GluN1/GluN2A-containing NMDARs and alters receptor function, a concept that has precedent in the reported interaction between the secondary conjugated bile acid chenodeoxycholic acid and NMDARs.^13^ Alternatively, GDCA may indirectly alter receptor function through downstream signaling processes. Further investigations into such a possibility would require targeted pharmacological and electrophysiological approaches, which were beyond the scope of the present study. In this regard, it is important to emphasize that our data indicate only an association between GDCA administration and reduced GluN1 and GluN2A subunits, BDNF mRNA levels, and CREB signaling. These findings do not provide mechanistic evidence that impairment of the NMDA-CREB-BDNF pathway underlies the observed deficits in recognition memory. Further functional experiments are therefore required to test this inferred model.

The lack of a significant effect of DCA on recognition memory in mice may be attributed to the differential FXR affinities of DCA and GDCA within the frontal cortex. Specifically, the ability of DCA to modulate FXR transcript and protein expression in the frontal cortex is consistent with its known affinity for this receptor, whereas GDCA appears unable to activate it.^31^ Our observed increase in cortical GluA1 phosphorylation following DCA administration suggests that the DCA activated FXRs, consistent with prior reports using FXR agonists.^12^ This may explain the subsequent reduction in the FXR mRNA and protein levels, which may be a homeostatic response to counterbalance excessive receptor activation. Nevertheless, in the absence of further functional studies, these changes remain correlative and do not establish a causal link to cognitive outcomes.

The observed reduction in GluN2A mRNA and protein in the frontal cortex following DCA administration suggests that DCA, like GDCA, has an FXR/TGR5-independent influence on NMDARs. However, DCA did not appear to alter CREB signaling. This could reflect a compensatory mechanism, where the DCA-induced disruption of NMDAR function, and the expected suppression of CREB activity were offset by an increase in GluA1 activity which is also known to enhance CREB signaling.^14^ Thus, although both DCA and GDCA reduced cortical GluN2A expression, only GDCA administration was associated with impaired cortical CREB-BDNF signaling and recognition memory deficits. A schematic representation of the proposed molecular events in the frontal cortex following DCA or GDCA exposure is presented in Figure S3 (Supplementary material). Earlier work has shown that FXR-mediated elevation of GluA1 phosphorylation is likely mediated by inhibition of the inflammasome, which is supported by our observation that DCA reduced IL-1β and IL-6 mRNA expression in the frontal cortex. It is reasonable to suggest, therefore, that stimulation of frontal cortex FXRs by DCA opposed its detrimental effects on NMDARs and maintained normal recognition memory. Of course, this scenario is speculative, and additional studies are required to further explore FXR-NMDAR-CREB interactions and DCA effects on frontal cortex function.

The reduction in spatial reference memory in the DCA group suggests that mice experienced greater difficulty in learning and recalling spatial cues necessary to identify the novel arm than control mice. Spatial working memory, assessed by spontaneous alternation, was unaffected by any of the bile acids. While the exact reason for this remains unclear, one possibility is that the mice had received bile acids for only 5 d at this stage of the experiment, which may have been too short a duration to affect working memory. Impaired CREB-BDNF signaling in the frontal cortex, a region essential for optimal working memory, was observed after 14 d of GDCA administration, leaving it uncertain whether a similar impairment was present when spontaneous alternation was assessed. Overall, these findings suggest that the relationship between bile acid signaling and different types of memory is complex and may involve distinct neural and temporal mechanisms that remain to be elucidated.

A decrease in TGR5 mRNA and protein and unaltered FXR mRNA and protein abundance observed in the hippocampus of the DCA-administered group aligns with the known higher affinity of DCA^32^ for TGR5. Additionally, the reduction in CREB phosphorylation suggests that DCA downregulated TGR5, thereby impairing CREB signaling and BDNF expression. This is in contrast to a previous study in which a TGR5 agonist administered over 5 d increased receptor levels and CREB activity.^11^ However, since DCA was administered for 14 d prior to tissue collection in our study, prolonged stimulation may have triggered negative feedback mechanisms, resulting in reduced TGR5 mRNA and encoding protein to prevent overstimulation. Therefore, it remains reasonable to propose that downregulation of hippocampal TGR5 reduces CREB-BDNF signaling, thereby impairing spatial reference memory. Impairments in spatial memory components have been previously linked to diminished CREB-BDNF pathways.^33^

It is worth noting that the differential effects of bile acids on receptor expression and signaling pathways in the frontal cortex and hippocampus may also be attributed to differences in the bioavailability of DCA and GDCA in the brain. To assess this, we administered plasma and brain bile acid levels approximately 2 h before the animals were culled. Although this time point was sufficient to detect circulating DCA and GDCA, their levels in the brain were very low, approaching the detection limit. This may suggest that two hours was insufficient for significant central accumulation, which might have been detectable at a later time point. Conversely, the accumulation of systemic bile acids in the brain might have been short-lived. Perino and colleagues^34^ reported that oral administration of a bile acid mimetic produced transient increases in hypothalamic concentrations, which declined within approximately 60 min, consistent with rapid clearance of bile acids from the brain. However, it is also important to note that peripheral bile acids might influence central receptor expression indirectly. For example, in a rodent model of acute liver failure, bile acids were observed to accumulate in the blood rather than the brain, yet the expression of FXR mRNA was reduced in the hippocampus.^35^ These findings highlight that observed changes in central bile acid receptors may reflect peripheral events rather than direct brain penetration.

Peripherally, however, bile acid metabolism appears to occur rapidly, as evidenced by the efficient deconjugation of administered GDCA to DCA (Table S1), indicating that the bile acids had reached the intestines and undergone microbial processing^36^ within this timeframe. Further investigation is needed to determine the timing and extent of bile acid penetration into the brain.

Although BSH gene abundance showed only a non-significant trend, the observed increase may have contributed to the rise in circulating DCA following GDCA administration. This interpretation remains tentative given the exploratory nature of the metagenomic analyzes and the small sample size (*n* = 6 per group). More broadly, we did not detect significant differences in overall microbial diversity or community composition among treatment groups, suggesting that neither DCA nor GDCA exposure induced substantial taxonomic restructuring of the gut microbiota. Likewise, untargeted functional profiling revealed only modest variation in microbial gene potential, with no effects surviving multiple-testing correction, consistent with the possibility that bile acid-related microbial changes were subtle and below the resolution of the current study. Together, these findings suggest that bile acid administration may influence microbial function more than community structure. However, larger, targeted studies are needed to confirm these trends and elucidate their mechanistic relevance. Importantly, we emphasize that these analyzes were exploratory, intended to assess whether there were homeostatic microbial responses that might counterbalance elevated bile acid levels, and that direct bile acid-microbiome interactions were beyond the scope of the study. Thus, the metagenomic analyzes were not performed to test whether the cognitive effects of DCA or GDCA were microbiome-mediated.

One potential limitation of the study is the uncertainty surrounding the central bioavailability of the administered bile acids. While DCA and GDCA were detected in plasma, their levels in the brain were near the detection limit, raising questions about whether the observed cognitive and molecular effects were due to direct central action or peripheral mechanisms. However, Perino and colleagues^34^ demonstrated that oral administration of a bile acid mimetic resulted in peak concentrations in the hypothalamus followed by a decline within 60 min, supporting the rapid clearance of bile acids from the brain. The observation that we detected changes in the levels of FXR and TGR5 in the cortex and hippocampus, respectively, further supports the notion that bile acids entered the brain, but were likely cleared well before the brain tissues were collected. Another limitation is that the short duration of bile acid exposure prior to the spontaneous alternation task (5 d) may have limited the ability to detect effects on working memory. It is possible, therefore, that a longer period of bile acid exposure would have been necessary to observe measurable changes in this cognitive function. Finally, mechanistic interpretations such as GDCA’s potential direct antagonism of NMDARs or DCA’s modulation of FXR and TGR5 signaling should be viewed as conceptual frameworks rather than explanatory conclusions. The present data demonstrate that exposure to these bile acids is associated with impaired recognition and spatial memory and coordinated changes in receptor and signaling encoding mRNAs and encoded proteins. However, they do not resolve whether these effects arise from direct receptor interactions, downstream signaling adaptations, or indirect systemic mechanisms. Discriminating between these possibilities will require targeted functional studies, which were beyond the scope of the present study.

In summary, this study provides novel evidence that the secondary bile acids, DCA and GDCA, differentially impair distinct domains of cognition, potentially through region- and receptor-specific mechanisms in the brain. GDCA selectively disrupted recognition memory, which was associated with reduced NMDAR subunit mRNA and protein expression and lower CREB-BDNF signaling in the prefrontal cortex, whereas DCA impaired spatial reference memory, and reduced TGR5 transcripts and protein and CREB activity in the hippocampus. These findings are correlative, and further studies are needed to establish causality. Furthermore, although these effects occurred without major alterations to the gut microbial community, subtle functional changes suggest possible bile acid-microbiota interactions that merit further exploration. Our findings collectively highlight bile acids as modulators of neuroplasticity and cognitive function, providing mechanistic insight into how peripheral metabolic disturbances may contribute to neurodegenerative processes and age-related cognitive decline. Future research should investigate the central bioavailability of these bile acids and utilize receptor-specific interventions to clarify causal pathways linking bile acid signaling to brain function.

## Supplementary Material

Garcia_Supplementary_MaterialR2 clean.docx Garcia_Supplementary_MaterialR2 clean.docx

## Data Availability

All data for this study can be openly accessed at the Oxford University Research Archive (ORA), DOI: 10.5287/ora-5r08j6x1e.
