# Supplementary material for: Glycodeoxycholic and deoxycholic bile acids impair recognition and spatial memory in adult mice, and reduce central CREB-BDNF signaling and cytokine expression with neuroanatomical specificity
Source: Gut Microbes. 2026 Jul 11;18(1):2701471. doi: 10.1080/19490976.2026.2701471 (PMC13360544; doi:10.1080/19490976.2026.2701471)
Supplement: Garcia_Supplementary_MaterialR2 clean.docx [file KGMI_A_2701471_SM5469.docx]

**SUPPLEMENTARY MATERIAL**

**METHODS**

**Bile acid quantification in mice brain and plasma**

*Sample preparation for mouse plasma and brain tissue*

Mouse blood plasma (50 µL) was thawed on ice and mixed with 150 µL of cold methanol and deuterated internal standards for bile acid quantification to precipitate proteins. The mixture was vortexed, incubated on ice for 15 minutes, and centrifuged at 15,000 × g for 10 minutes at 4°C. Supernatants of a fixed volume were collected for LC-MS analysis.

Frozen mouse brain tissue (~15 mg) was transferred directly into pre-filled ceramic bead tubes and homogenized using a FastPrep®-24 Classic bead beating grinder and lysis system. Homogenization was performed for 30 seconds at a set speed, repeated five times with resting intervals of 30 seconds each. Protein precipitation was achieved by adding ice-cold methanol and dueterated internal standards to the homogenate, followed by vortexing and incubation on ice for 15 minutes. Samples were centrifuged at 15,000 × g for 10 minutes at 4°C. A fixed amount of the clear supernatant was collected for direct LC-MS injection (5 µL).

*UPLC parameters*

Bile acid separation was performed using a Waters BEH C8 column (100 mm × 2.1 mm, 1.7 µm) maintained at 60°C. Mobile phase A consisted of water and acetonitrile (10:1, v/v) with 0.007% ammonium acetate and 0.0318% acetic acid (pH 4.16), while mobile phase B was acetonitrile and isopropyl alcohol. The flow rate was initially 0.6 mL/min, increased gradually to 1 mL/min during the run, and returned to initial conditions by the end. Gradient elution was set as follows: 10% B at 0 min, ramped to 35% B at 9.25 min, 85% B at 11.25 min, 100% B at 11.8 min, held until 13.05 min, then returned to 10% B at 13.1 min and maintained until 18 min.

*Xevo TQ-XS parameters*

Key MS settings included a capillary voltage of 1.57 kV, cone voltage of 60 V, source offset of 30 V, desolvation temperature at 600°C, source temperature at 150°C, desolvation gas flow of 1000 L/hr, cone gas flow of 150 L/hr, and nebulizer gas pressure of 7.0 bar. Multiple reaction monitoring (MRM) transitions tailored for specific bile acids were employed. Compound-specific collision energy values and MRM transitions followed a previously validated protocol (Sarafian et. al., 2015)

*Data analysis*

Raw data were processed using Waters MassLynx software v4.2 (package: TargetLynx). Bile acid concentrations were determined by calculating the integrated peak area ratios of analytes to their corresponding internal standards. Quantification employed a linear regression model applying a 1/x weighting factor. Calibration curves were validated to ensure linearity with R^2^ ≥ 0.99 and accuracy within 20% of QC sample values. Quantitated bile acid concentrations were used for downstream statistical analysis. Missing values and outliers were addressed as appropriate.

**Mouse faecal metagenomic analysis**

*Sequencing and data processing*

Transnetyx Microbiome kits containing barcoded sample collection tubes were provided by Transnetyx (Cordova, TN, USA). Mouse fecal samples (6 samples per group) were placed in individual tubes containing DNA stabilization buffer to ensure reproducibility, stability, and traceability, and shipped for DNA extraction, library preparation, and sequencing by Transnetyx (Cordova, TN USA). DNA extraction was optimized and fully automated using a robust process for reproducible extraction of inhibitor-free, high molecular weight genomic DNA that captures the true microbial diversity of stool samples. After DNA extraction and quality control (QC), genomic DNA was converted into sequencing libraries using a method optimized for minimal bias. Unique dual indexed (UDI) adapters were used to ensure that reads and/or organisms are not mis-assigned. After QC, the libraries were sequenced using the shotgun sequencing method (a depth of 2 million 2x150 bp read pairs), which enables species and strain level taxonomic resolution. Sequencing was performed using the Illumina NextSeq 2000 instrument and protocol.

Raw data (in the form of FASTQ files) were uploaded automatically onto One Codex analysis software and analyzed against the One Codex database consisting of >148K whole microbial reference genomes. The classification results were filtered through several statistical post-processing steps designed to eliminate false positive results caused by contamination or sequencing artifacts. The One Codex Database consists of ~148K complete microbial genomes, including 71K distinct bacterial genomes, 72K viral genomes, and thousands of archaeal and eukaryotic genomes. Human and mouse genomes are included to screen out host reads. The database is assembled from both of public and private sources, with a combination of automated and manual curation steps to remove low quality or mislabeled records.

One Codex functional analysis leverages the [Humann3](http://huttenhower.sph.harvard.edu/humann) pipeline. It analyzes whole-genome shotgun sequencing data to identify genes, profiling their function and contribution to metabolic pathways. The first step characterizes the species composition of the sample. Traditionally, this step utilizes [MetaPhlAn](https://github.com/biobakery/MetaPhlAn), however the One Codex functional analysis has been modified to use the [One Codex database](https://docs.onecodex.com/en/articles/3761205-one-codex-database) and [metagenomic classifier](https://docs.onecodex.com/en/articles/3753847-metagenomic-classification). After classification, reads are mapped to a custom annotated database of those species’ pangenomes to identify gene families and taxonomy (with bowtie2 + ChocoPhlAn). It further does translated search on any unmapped reads (using Diamond and the UniRef90 database) to identify gene families.

*Diversity and Functional Analyses*
Within-sample microbial diversity (alpha diversity) was assessed using observed richness, Shannon, and Simpson indices, calculated in R with *vegan*. Differences in community composition between samples (beta diversity) were quantified using Bray-Curtis dissimilarities and visualized via principal coordinate analysis (PCoA), with group-level effects evaluated using PERMANOVA (*vegan*). Differential abundance of microbial taxa was examined with *DESeq2*, which models count data using a negative binomial distribution and adjusts for multiple testing using the Benjamini-Hochberg procedure. Microbial functional potential, expressed as copies per million (CPM), was explored using PCA (*stats*) to assess overall variation among groups, and pairwise Wilcoxon rank-sum tests (*stats*) were performed to identify gene-level differences, with FDR correction applied. Volcano plots were generated (*ggplot2*) to highlight both the magnitude (log₂ fold change) and statistical significance of functional gene changes, including targeted analyses of hydrolase genes potentially linked to bile acid metabolism.

**RESULTS**

***Bile acid concentrations in plasma***

| **GROUP**  **(male/female)** | **PLASMA BILE ACID [nM] (Mean +/- SEM)** | | |
| --- | --- | --- | --- |
|  | **Deoxycholic acid** | **Glycodeoxycholic acid** | **Cholic acid** |
| Control (6/6) | 573.6 + 173.8 | 0.8 + 0.7 | 243.7 + 69.8 |
| DCA (4/5) | 3649.5 + 576.6***** | 0.4 + 0.3 | 5711.1 + 3235.8***** |
| GDCA (6/6) | 3512.3 + 950.0***** | 4162.4 + 2120.9* | 13063.7 + 4857.0***** |

**Table S1.** The effect of an oral administration of deoxycholic acid (DCA) or glycodeoxycholic acid (GDCA) on plasma bile acid concentrations in male and female mice measured by LC-MS. All data were analysed using the Kruskal-Wallis test, followed by post hoc between-group comparisons with Mann-Whitney U tests when significant differences were detected.*P<0.001 compared to the control group. Control, n=12; DCA, n=9; GDCA, n=12.

***Metagenomic functional analysis***

*(i) Targeted analysis of Bile Salt Hydrolase (BSH).*


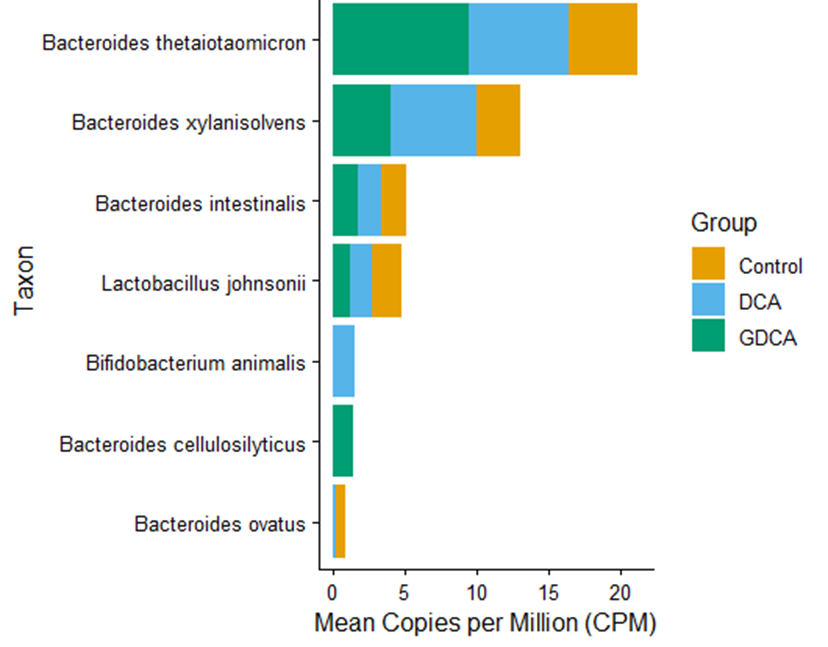


**A**

**B**

**Figure S1**. Targeted analysis of bile salt hydrolase (BSH) activity in faecal samples from control and bile acid-fed mice. **A**) Relative abundance of the microbial BSH gene expressed as copies per million (CPM). Kruskal-Wallis analysis revealed a trend toward a difference between groups (*H* = 5.099, *df* = 2, *p*=0.078). **B**) Taxonomic contributors to BSH activity across the control and bile acid-fed mice. Horizontal stacked bars show the mean abundance of BSH-associated taxa, expressed as mean CPM. Each bar represents the cumulative contribution of the indicated taxon to total BSH abundance across groups. Unclassified taxa were excluded. BSH abundance was primarily associated with *Bacteroides spp.*, with additional contributions from *Lactobacillus johnsonii* and *Bifidobacterium animalis*. This BSH distribution pattern has been reported in the human gut (Song et al., 2019)

*(ii) Untargeted analysis of ‘Hydrolase’ function*

A PCA of hydrolase-related gene abundances (expressed as CPM) revealed overlapping clustering among groups, indicating no distinct separation in functional profiles (Fig S1A). To explore potential microbial mechanisms linked to the observed increase in circulating cholic acid and DCA after bile acid administration (see Table S1), we examined differential abundance of genes annotated with hydrolase activity. Volcano plots comparing DCA and GDCA groups to controls revealed that no hydrolase-related functions were significantly altered in the DCA group (Fig S1B). In contrast, the GDCA group showed modest enrichment of several hydrolase functions, including *hydrolase activity acting on carbon–nitrogen (but not peptide) bonds* and *sulfuric ester hydrolase activity* (Fig S1C).

To identify the potential drivers of functional change, we examined *sulfuric ester hydrolase activity* as it demonstrated the strongest trend toward significance. The top taxa contributing to this function were identified and plotted (Fig S1D). This exploratory analysis revealed that members of the *Bacteroides* genus, along with *Akkermansia muciniphila* and *Parabacteroides goldsteinii*, contributed most strongly to this activity, with the highest relative abundance observed in the GDCA group.

*Abundance analysis*

Nonparametric analysis of the abundance of bacterial species associated with sulphuric hydrolase activity in all groups, revealed a significant group difference in *Bacteroides intestinalis* levels (Table S2). There was also a trend (p=0.055) group difference in *Akkermansia munciniphila.*  However, nominal differences between groups did not survive FDR correction

**Table S2.** The effect of an oral administration of deoxycholic acid (DCA) or glycodeoxycholic acid (GDCA) on relative abundance (counts per million, CPM) of bacterial species associated with sulphuric hydrolase activity. All data were analysed using the Kruskal-Wallis test. N= 6 (3 male, 3 female) per group. Species with nominal significance (p < 0.05) are indicated, but none remained significant after FDR correction.

| Species | GROUP (CPM + SEM) | | | Kruskal-Wallis  H-value | Kruskal-Wallis  p-value |
| --- | --- | --- | --- | --- | --- |
|  | Control | DCA | GDCA |  |  |
| *Akkermansia muciniphila* | 22 + 14 | 42 + 27 | 82 + 20 | 5.80 | 0.055 |
| *Bacteroides xylanisolvens* | 8 + 2 | 16 + 4 | 12 + 2 | 3.61 | 0.164 |
| *Bacteroides thetaiotaomicron* | 11 + 5 | 18 + 5 | 20 + 7 | 1.98 | 0.372 |
| *Parabacteroides goldsteinii* | 33 +12 | 39 + 17 | 30 + 4 | 0.19 | 0.911 |
| *Bacteroides ovatus* | 39 + 17 | 70 +18 | 75 + 16 | 3.61 | 0.164 |
| *Bacteroides intestinalis* | 4 + 2 | 16 + 5 | 19 + 4 | 7.52 | **0.023** |
| *Bacteroides caecimuris* | 15 + 10 | 7 + 4 | 4 + 3 | 1.08 | 0.582 |
| *Lactobacilus johnsonii* | 15 + 13 | 4 + 3 | 2 + 1 | 0.32 | 0.853 |
| *Bifidobacterium animalis* | 6 + 3 | 12 + 6 | 7 + 2 | 1.34 | 0.512 |
| *Bacteroides cellulosyliticus* | 7 + 4 | 13 + 8 | 4 + 3 | 0.86 | 0.650 |
| *Muribaculum intestinale* | 3 + 2 | 8 + 4 | 12 + 2 | 4.04 | 0.132 |


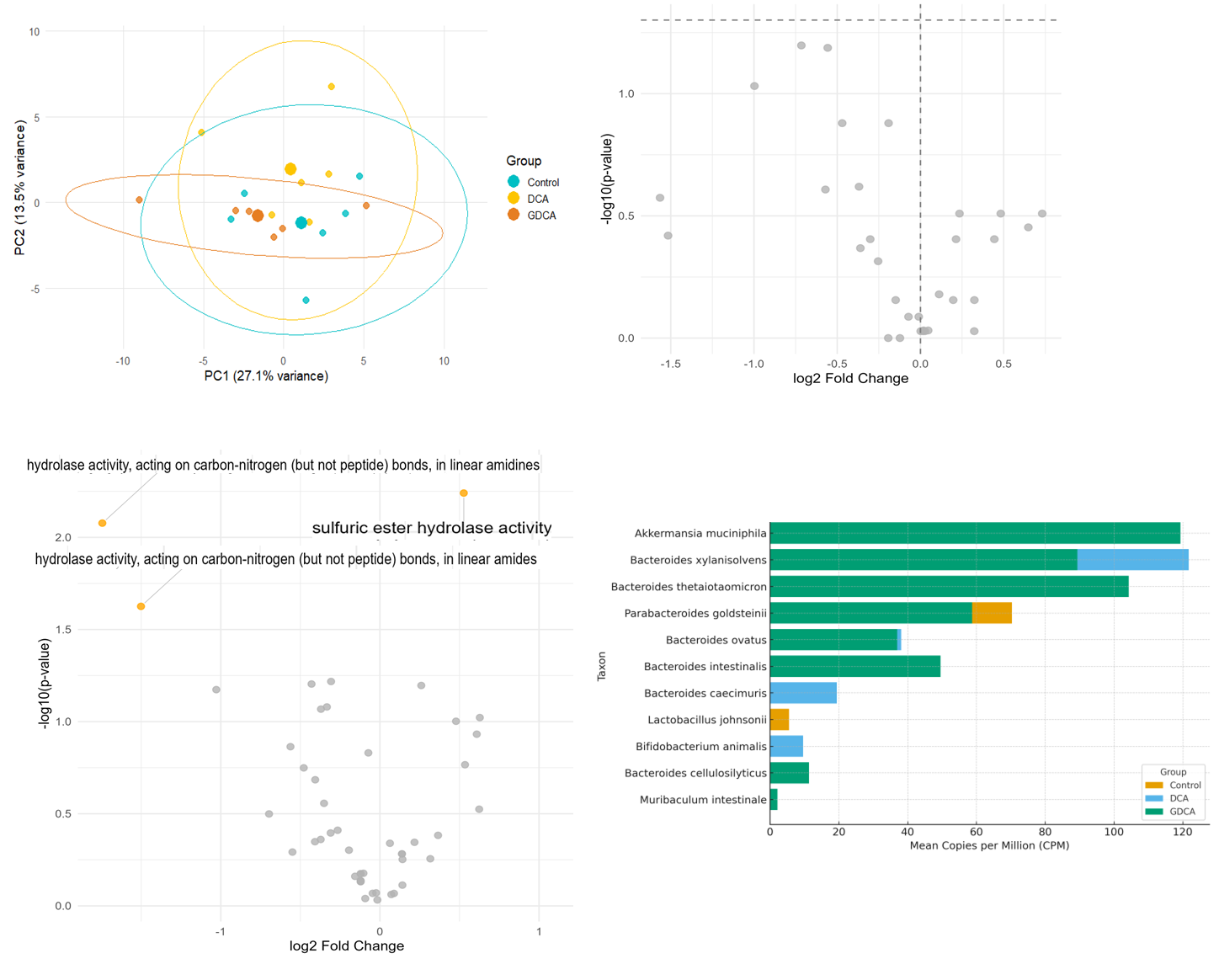


**A**

**B**

**D**

**C**

**Figure S2.** Microbial community structure and functional analysis under bile acid treatments.
(**A**) Principal Component Analysis (PCA) of microbial community composition across Control, DCA, and GDCA treatment groups. Each point represents an individual sample, and ellipses denote 95% confidence intervals for each group. Larger points indicate group centroids representing the average community composition within each treatment. (**B**) Volcano plot showing differential microbial taxa between the Control and DCA groups. Grey points represent non-significant features, while orange points indicate features with unadjusted significance (*p* < 0.05). The x-axis shows the log₂ fold change, and the y-axis represents the –log₁₀(*p*) value. (**C**) Volcano plot showing differential microbial taxa between the Control and GDCA groups. Grey points represent non-significant features, and orange points denote features with unadjusted significance (*p* < 0.05). Significantly enriched enzyme activities include hydrolase activity acting on carbon–nitrogen (non-peptide) bonds and sulphuric ester hydrolase activity. (**D**) Relative abundance of dominant bacterial taxa (mean copies per million, CPM) across groups. Bars represent the contribution of each group to sulphuric ester hydrolase activity.

**
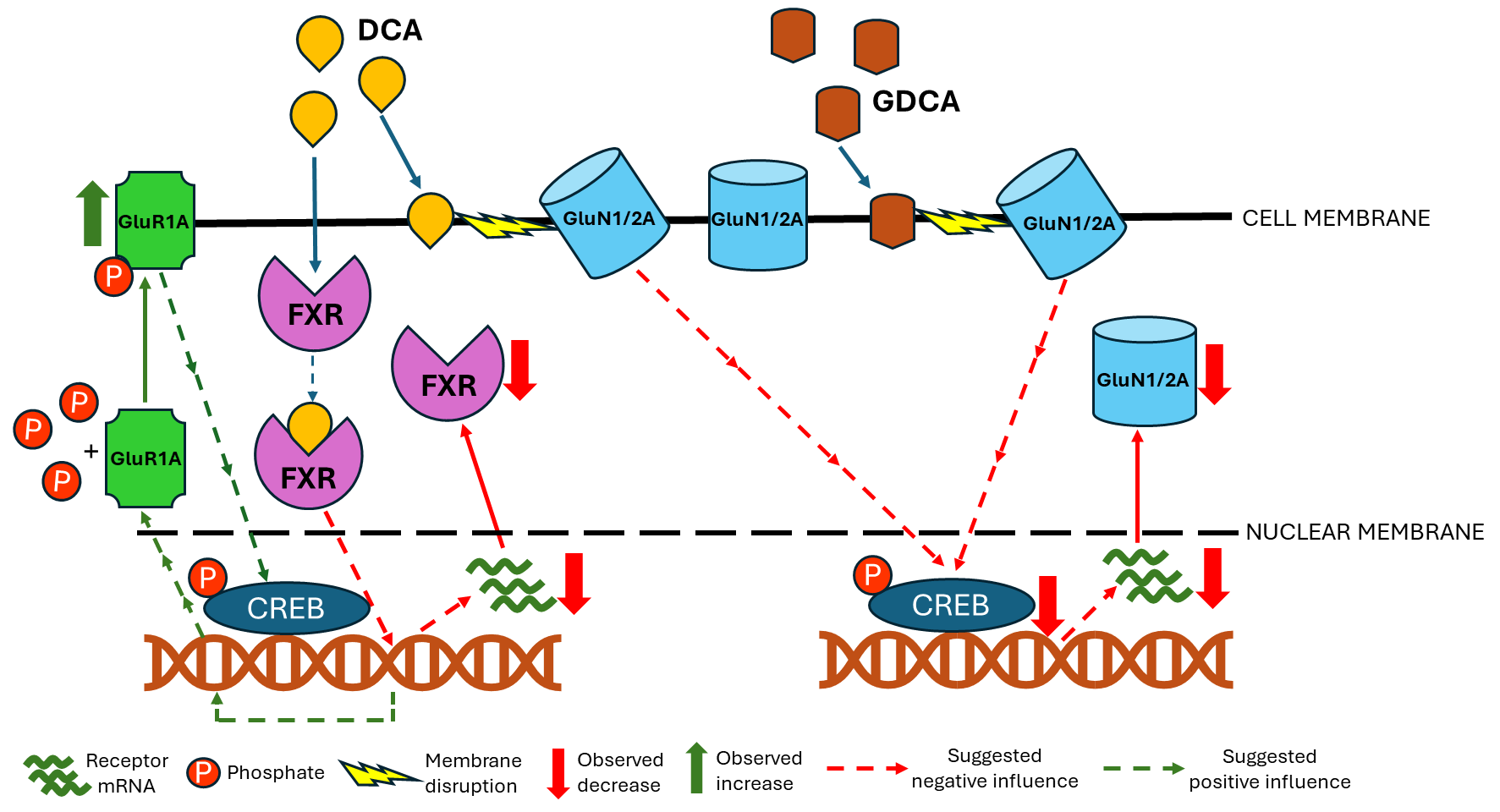
**

**Figure S3. Proposed model of DCA- and GDCA-mediated modulation of receptor expression and CREB signalling in the frontal cortex.** **DCA intake** reduces **FXR mRNA and protein abundance, which is suggested as a negative feedback response (illustrated by red dashed arrows) to counter-balance excessive DCA concentrations in the brain.** DCA administration, through its presumed binding to FXR, is proposed to promote phosphorylation of AMPA receptor GluA1 subunits and their subsequent insertion into the neuronal cell membrane. The resulting increase in AMPA receptor activity is suggested to enhance CREB signalling (illustrated by green dashed arrows). At the same time, extracellular DCA is proposed to disrupt the membrane localisation of nearby GluN2A-containing NMDA receptors as the bile acid diffuses into the cell. This disruption reduces GluN2A/NMDA receptor activity, leading to decreased CREB phosphorylation and consequently reduced GluN2A expression. Although NMDA receptor mediated CREB activation is therefore diminished, the increased phosphorylation of GluA1 and the resulting enhancement of AMPA receptor signalling are proposed to compensate for this reduction. As a result, overall CREB activity is predicted to remain unchanged in the frontal cortex following DCA administration. This balance between reduced NMDA receptor signalling and increased AMPA receptor signalling may explain why CREB levels are not altered and why recognition memory remains normal in DCA administered mice. **GDCA intake** has a lower affinity for FXR but, like DCA, its diffusion into the cell disrupts membrane localisation of nearby GluN2A-containing NMDA receptors reduces GluN2A/NMDA receptor activity, leading to decreased CREB phosphorylation and consequently reduced GluN2A expression. As a consequence, GDCA is proposed to cause a similar destabilisation of NMDA receptors as DCA, which is reflected not only in alterations to GluN2A but also in significant reductions in GluN1 mRNA and protein abundance. Unlike DCA, this disruption is not compensated by AMPA receptor activation and so is assumed to underlie the observed impairment of recognition memory following GDCA administration. Solid arrows indicate changes observed with QPCR and western blot measurements.

**Supplementary References**

Sarafian MH, Lewis MR, Pechlivanis A, Ralphs S, McPhail MJW, Patel VC, Dumas M-E, Holmes E, Nicholson JK (2015). Bile Acid profiling and quantification in biofluids using Ultra-Performance Liquid Chromatography Tandem Mass Spectrometry. *Analytical Chemistry*. 87(19):9662-9670.

Song Z, Cai Y, Lao X, Wang X, Lin X, Cui Y, Kalavagunta PK, Liao J, Jin L, Shang J, Li J. (2019). Taxonomic profiling and populational patterns of bacterial bile salt hydrolase (BSH) genes based on worldwide human gut microbiome. *Microbiome.* 7(1):9.
